# Placental Growth Factor Secreted from Placenta-Derived Mesenchymal Stem Cells Improves Ovarian Function in TAA-Injured Rats via Antioxidant Effects

**DOI:** 10.3390/antiox15050603

**Published:** 2026-05-10

**Authors:** Jae-Young Shin, Dae-Hyun Lee, Hyeri Park, Young Ran Kim, Gi Jin Kim

**Affiliations:** 1 Department of Biomedical Science, CHA University, Pocheon 11160, Republic of Korea; shjy0107@gmail.com (J.-Y.S.); hyeyeyeri@gmail.com (H.P.); 2 Department of Convergence Science, CHA University, Pocheon 11160, Republic of Korea; ldh1532@chauniv.ac.kr; 3 Research Institute of Placental Science, CHA University, Pocheon 11160, Republic of Korea; 4 Department of Obstetrics and Gynecology, CHA Bundang Medical Center, CHA University, Pocheon 11160, Republic of Korea; happyiran@cha.ac.kr

**Keywords:** ovarian dysfunction, metabolic disorder, PlGF, placenta-derived mesenchymal stem cells

## Abstract

Ovarian dysfunction resulting from metabolic or toxic injury is characterized by follicular depletion, stromal remodeling, oxidative stress, and endocrine dysregulation. Placenta-derived mesenchymal stem cells (PD-MSCs) have been proposed as a potential therapeutic approach due to their paracrine factors, including placental growth factor (PlGF). However, the pathways through which PD-MSCs exert protective effects on the ovary remain insufficiently defined. In this study, we examined whether PD-MSC transplantation ameliorates ovarian injury in a thioacetamide (TAA)-induced ovarian insufficiency model and explored the signaling events potentially associated with this response. Female rats were administered TAA for 12 weeks, and PD-MSCs were transplanted at week 8. We assessed ovarian morphology, fibrosis, oxidative stress markers, hormonal profiles, and follicle development. Complementary in vitro experiments using TAA-treated KGN granulosa-like cells were performed to investigate potential mechanistic associations. PD-MSC transplantation improved ovarian architecture, reduced collagen deposition, enhanced follicle growth, and mitigated oxidative stress. These changes were accompanied by increased PlGF expression and enhanced activation of fms-like tyrosine kinase-1 (Flt-1), p38 mitogen-activated protein kinase (p38 MAPK), extracellular signal-regulated kinase (ERK), and nuclear factor erythroid 2-related factor 2 (Nrf2)-related antioxidant pathways. In vitro, PD-MSCs coculture similarly attenuated oxidative stress and partially improved mitochondrial membrane potential in damaged KGN cells. Together, these findings suggest that PD-MSCs ameliorate ovarian structural damage and oxidative stress in TAA-induced injury, potentially through paracrine mechanisms partly involving PlGF/Flt-1-associated antioxidant signaling. This work supports the therapeutic potential of PD-MSCs for metabolic or toxicant-induced ovarian insufficiency while underscoring the need for further studies to fully delineate the specific contribution of PlGF and its interaction with downstream antioxidant pathways.

## 1. Introduction

Oxidative stress, defined as an imbalance between reactive oxygen species (ROS) production and antioxidant defenses, is a key mediator of ovarian injury. Excessive accumulation of ROS creates a vicious cycle of reduced antioxidant capacity and persistent inflammation, contributing to ovarian dysfunction and reproductive aging. In the ovary, elevated ROS levels induce lipid peroxidation, mitochondrial dysfunction, and granulosa cell apoptosis, ultimately impairing oocyte maturation and accelerating follicular atresia [1]. In addition to direct cellular damage, ROS promote extracellular matrix (ECM) remodeling and stromal fibrosis, further suppressing follicular development and compromising ovarian function [2,3]. Accordingly, oxidative stress is now recognized as a central mechanistic link between metabolic imbalance and progressive reproductive decline.

Ovarian dysfunction arises when ovarian homeostasis is disrupted by hormonal imbalance, metabolic dysregulation, or inflammation, resulting in impaired folliculogenesis, diminished ovarian reserve, and ovulation failure [4]. Among ovarian disorders, polycystic ovary syndrome (PCOS) is closely associated with metabolic disturbances such as insulin resistance, dyslipidemia, and obesity. These metabolic abnormalities lead to endocrine imbalance, abnormal follicular growth, reduced oocyte quality and elevated levels of luteinizing hormone (LH) and testosterone [5]. Moreover, animal and clinical studies have shown that metabolic overload, particularly lipid accumulation and insulin resistance, exacerbates oxidative damage in ovarian cells, promoting follicular apoptosis and impairing hormone secretion [2,4]. Together, these findings highlight the close interplay between metabolic dysfunction and oxidative stress in the progression of ovarian dysfunction.

Current treatment strategies for ovarian dysfunction often involve hormone replacement therapy (HRT) or mesenchymal stem cells (MSCs) derived from various tissues (e.g., adipose tissue, bone marrow, or the placenta) [6]. Although HRT can alleviate symptoms and temporarily correct hormone imbalances, its long-term use is associated with increased risks of cardiovascular disease and cancer and does not address underlying follicular damage or ROS-related damage [7]. MSC-based therapies have shown promise in preclinical models for restoring ovarian structure and function. However, several limitations remain, including low homing efficiency, variability in paracrine factor secretion, donor-dependent heterogeneity, and ethical or invasive concerns related to tissue harvesting [8,9].

PD-MSCs have recently attracted attention as a potential alternative. They can be readily obtained from discarded placental tissue and exhibit lower immunogenicity, more consistent secretion of paracrine factors, and greater proliferative capacity than adipose tissue-derived MSCs (AD-MSCs) or bone marrow-derived MSCs (BM-MSCs) in several studies [10,11]. These properties may make PD-MSCs particularly effective in overcoming oxidative and inflammatory microenvironments associated with ovarian injury.

One key paracrine molecule secreted by PD-MSCs is placental growth factor (PlGF). PlGF belongs to the vascular endothelial growth factor (VEGF) family and preferentially binds to Fms-like tyrosine kinase-1 (Flt-1; VEGFR-1), thereby regulating angiogenesis, inflammation, and cell survival under stress conditions [12]. In several pathological states, including PCOS and ischemic injury, PlGF expression has been reported to be reduced, whereas restoration of PlGF levels has been linked to improved vascular remodeling, decreased ROS levels, and enhanced tissue regeneration [13,14]. The functional roles of PlGF have been increasingly recognized, particularly in the context of disease progression [15]. In addition to its role in PCOS, PlGF imbalance has been implicated in other pathological conditions. For instance, in preeclampsia, elevated levels of the soluble decoy receptor sFlt-1 disrupt the balance between PlGF and sFlt-1, thereby reducing PlGF bioavailability [16].

PlGF exerts its effects primarily by binding to Flt-1, initiating downstream phosphorylation events that activate the p38 mitogen-activated protein kinase (p38 MAPK) and extracellular signal-regulated kinase (ERK) pathway [17,18]. These kinases have been reported to regulate the nuclear translocation of nuclear factor erythroid 2-related factor 2 (Nrf2), a master regulator of cellular redox homeostasis [19]. Activated Nrf2 binds to antioxidant response elements and induces the transcription of antioxidant enzymes, including heme oxygenase-1 (HO-1), Catalase, and peroxiredoxins, thereby mitigating oxidative stress and preserving tissue integrity [20]. In pathological ovaries, however, this signaling cascade is attenuated. Reduced p38/ERK activity in granulosa cells is associated with impaired cell survival and defective follicle maturation [21,22]. Similarly, decreased nuclear Nrf2 expression and reduced levels of its target enzymes are consistent with an oxidative environment that promotes follicular atresia and stromal remodeling [5]. These observations support the concept that suppression of the PlGF/Flt-1-p38 MAPK/ERK/Nrf2 axis contributes to ovarian injury [23,24] and suggest that modulation of this signaling axis may represent a potential therapeutic target for PD-MSC-mediated effects.

Although our previous report demonstrated that PD-MSCs exert antioxidant effects in TAA-induced ovarian injury, the underlying signaling mechanisms remain incompletely defined [25,26]. In this study, we hypothesized that PD-MSC transplantation attenuates TAA-induced ovarian injury through paracrine mechanisms potentially involving PlGF/Flt-1-associated antioxidant pathways. We further aimed to evaluate morphological, molecular, and redox-related alterations in ovarian tissues following PD-MSC transplantation using complementary in vivo and in vitro approaches.

## 2. Materials and Methods

### 2.1. Cell Culture

Human placental tissues were collected from women at term (approximately 37 weeks of gestation) with no obstetric or perinatal complications. All procedures were approved by the Institutional Review Board of CHA General Hospital, Seoul, Republic of Korea (IRB-07-18), and written informed consent was obtained from all participants prior to sample collection. PD-MSCs were isolated from the chorionic plate as described previously [27] and maintained in alpha-modified minimum essential medium (α-MEM; HyClone, Logan, UT, USA) supplemented with 10% fetal bovine serum (FBS; Gibco, Carlsbad, CA, USA), 1% penicillin/streptomycin (P/S; Gibco), 25 ng/mL human fibroblast growth factor-4 (hFGF-4; PeproTech, Rocky Hill, NJ, USA), and 1 μg/mL heparin (Sigma-Aldrich, St. Louis, MO, USA) at 37 °C in a humidified atmosphere with 5% CO_2_. Before transplantation, the PD-MSCs were labeled with a PKH67 Fluorescent Cell Linker Kit (Sigma-Aldrich) according to the manufacturer’s instructions, and 2 × 10^6^ cells were suspended in sterile PBS for intravenous administration to the rats in the transplantation group. For comparative analysis of PlGF secretion, WI-38 fibroblasts were cultured in α-MEM supplemented with 10% FBS and 1% P/S; BM-MSCs were maintained in α-MEM supplemented with 10% FBS, 4 mM L-glutamine, and 1% P/S; and AD-MSCs were cultured in α-MEM supplemented with 10% FBS, 4 mM L-glutamine, and 1% P/S. All cell types were expanded to passage 8, seeded and cultured for 4 days under the above conditions, and one day prior to harvest, the medium was replaced with formulations containing serum-free α-MEM to minimize the influence of exogenous serum-derived factors; the conditioned media were subsequently collected and used for PlGF quantification by ELISA.

### 2.2. Human Granulosa-like KGN Tumor Cells

The human granulosa-like tumor cell line KGN was obtained from Riken BioResource Center (Saitama, Japan). KGN cells were cultured in Dulbecco’s modified Eagle’s medium/Ham’s F-12 (DMEM/F12; Gibco, Carlsbad, CA, USA) supplemented with 10% FBS. The cultures were maintained at 37 °C in a humidified incubator containing 5% CO_2_. For the in vitro oxidative injury modeling, KGN cells were exposed to 70 mM TAA (Sigma-Aldrich, St. Louis, MO, USA) for 24 h to induce oxidative stress. Untreated KGN cells served as negative controls, whereas TAA-treated KGN cells without PD-MSC coculture served as injury models. Following TAA exposure, KGN cells were co-cultured with 5 × 10^4^ PD-MSCs seeded in Transwell inserts (0.8 µm pore size; Corning, NY, USA) for 24 h, representing the experimental treatment condition. After co-culture, KGN cells were harvested for analysis. All in vitro experiments using KGN cells were performed in three independent biological replicates (*n* = 3) unless otherwise specified.

### 2.3. Small Interfering RNA Transfection

For transfection, PD-MSCs were seeded at an appropriate density and allowed to adhere overnight under standard culture conditions as described above. On the day of transfection, PlGF-targeting siRNA (20 nmol; Bioneer, Daejeon, Republic of Korea; sense: 5-CUGAGAAGAUGCCGGUCAU-3, overhang: dTdT, antisense: 5-AUGACCGGCAUC UUCUCAG-3, overhang: dTdT) and Lipofectamine 2000 (Invitrogen, Carlsbad, CA, USA) were separately diluted in Opti-MEM reduced-serum medium (Gibco, Waltham, MA, USA). The diluted Lipofectamine 2000 was incubated for 5 min at room temperature, after which it was combined with the diluted siPlGF and allowed to form lipid-RNA complexes for an additional 5 min. The resulting transfection mixture was then added to the PD-MSCs. Following transfection, PD-MSCs were incubated for 24 h at 37 °C in a humidified atmosphere containing 5% CO_2_ to allow effective PlGF knockdown. Transfected PD-MSCs were subsequently used for analyses. For in vitro coculture assays, KGN cells were first exposed to TAA for 24 h to induce cellular stress. After TAA treatment, KGN cells were cocultured with PD-MSCs using a Transwell system. During coculture, PD-MSCs were transfected with PlGF-targeting siRNA, whereas control PD-MSCs received no transfection. Cells and conditioned media were collected 24 h after coculture initiation for analyses.

### 2.4. TAA Animal Model and PD-MSC Transplantation

All animal experiments were approved by the Institutional Animal Care and Use Committee of CHA University, Seongnam, Republic of Korea (IACUC-2400016). Female Sprague–Dawley rats (7 weeks old; Orient Bio Inc., Seongnam, Republic of Korea) were housed in a pathogen-free facility under controlled environmental conditions (21 ± 1 °C, 12 h light/dark cycle, and ad libitum access to standard chow and water). Animals were randomly assigned into three groups: normal control (Nor, *n* = 6), TAA-treated group without PD-MSCs transplantation (NTx, *n* = 8), and TAA-treated group receiving PD-MSCs transplantation (Tx, *n* = 6). The Nor group served as the negative control, whereas the NTx group served as the injury positive control for all in vivo analyses. To induce ovarian injury, 150 mg/kg TAA (Sigma-Aldrich, St. Louis, MO, USA) was administered intraperitoneally twice per week for a total of 12 weeks. During week 8 of TAA treatment, placenta-derived mesenchymal stem cells (PD-MSCs; 2 × 10^6^ cells in sterile PBS) were transplanted via the tail vein. Animals were maintained until week 12, at which point they were sacrificed, and ovarian tissues were collected for subsequent analyses. All in vivo procedures were conducted in accordance with the ARRIVE 2.0 (Animal Research: Reporting of In Vivo Experiments) guidelines [28] to ensure methodological rigor, reproducibility, and adherence to ethical standards in animal research.

### 2.5. RNA Isolation and Quantitative Real-Time Polymerase Chain Reaction

Ovarian tissues were first homogenized in liquid nitrogen, and total RNA was extracted using TRIzol reagent (Ambion, Thermo Fisher Scientific, Waltham, MA, USA) according to the manufacturer’s guidelines. RNA concentration and purity were assessed with a NanoDrop spectrophotometer (Thermo Fisher Scientific, Waltham, MA, USA). Complementary DNA (cDNA) was synthesized from the isolated RNA using Superscript III reverse transcriptase (Invitrogen, Waltham, MA, USA). The reverse transcription program consisted of incubation at 65 °C for 5 min, 4 °C for 1 min, 50 °C for 60 min, and a final step at 72 °C for 15 min. Quantitative real-time PCR (qRT-PCR) was performed using SYBR Ex Taq (Roche, Basel, Switzerland). Amplification was carried out with initial denaturation at 95 °C for 5 s, followed by 50 cycles of 95 °C for 5 s and 60 °C for 30 s. The sequences of the qRT-PCR primers used are listed in Supplementary Table S1. GAPDH served as an internal control for normalization, and each sample was analyzed in triplicate. RNA samples were obtained from the same animal cohorts described in the TAA model section (Nor; *n* = 6, NTx; *n* = 8, Tx; *n* = 6).

### 2.6. Protein Isolation and Western Blot Analyses

Ovarian tissues from each experimental group were homogenized in liquid nitrogen and lysed using radioimmunoprecipitation assay (RIPA) buffer (Sigma-Aldrich, St. Louis, MO, USA) supplemented with a protease inhibitor cocktail (Roche, Basel, Switzerland) and a phosphatase inhibitor cocktail (genDEPOT, Katy, TX, USA). Protein concentrations were determined using a bicinchoninic acid (BCA) protein assay kit (Thermo Fisher Scientific, Waltham, MA, USA), and equal amounts of protein were loaded into each sample. Lysates were separated by 8–12% sodium dodecyl sulfate–polyacrylamide gel electrophoresis (SDS–PAGE) and transferred to polyvinylidene difluoride (PVDF) membranes (Bio-Rad Laboratories, Hercules, CA, USA) using a Turbo Transfer system (Bio-Rad Laboratories). After transfer, the membranes were washed with Tris-buffered saline containing 0.1% Tween-20 (TBS-T) for 5 min and blocked with 5% bovine serum albumin (BSA; Sigma-Aldrich) in TBS-T for 1 h at room temperature. The membranes were subsequently incubated overnight at 4 °C with primary antibodies diluted in 2% BSA. The following primary antibodies were used: rabbit anti-TGF-β1 (ab92486, Abcam, Cambridge, UK; 1:1000), goat anti-SMAD2/3 (SC-6032, Santa Cruz Biotechnology, Dallas, TX, USA; 1:1000), mouse anti-SMAD4 (SC-7966, Santa Cruz Biotechnology; 1:1000), mouse anti-α-SMA (ab7817, Abcam; 1:1000), mouse anti-Collagen I (MA1-26771, Invitrogen, Carlsbad, CA, USA; 1:1000), rabbit anti-PlGF (bs-0280R, Bioss, Woburn, MA, USA; 1:1000), rabbit anti-Flt-1 (NBP3-11864, Novus Biologicals, Centennial, CO, USA; 1:500), rabbit anti-phospho-p38 MAPK (4511S, Cell Signaling Technology, Danvers, MA, USA; 1:1000), rabbit anti-total p38 MAPK (8690S, Cell Signaling Technology; 1:1000), mouse anti-phospho-ERK (9106S, Cell Signaling Technology; 1:1000), rabbit anti-total ERK (4695S, Cell Signaling Technology; 1:1000), rabbit anti-phospho-Nrf2 (bs-2013R, Bioss; 1:1000), rabbit anti-total Nrf2 (bs-1074R, Bioss; 1:1000), mouse anti-HO-1 (NBP1-97597, Novus Biologicals; 1:500), rabbit anti-Prx2 (MA5-32468, Invitrogen, Carlsbad, CA, USA; 1:1000), rabbit anti-Catalase (ab52477, Abcam; 1:1000), rabbit anti-BMP15 (MBS2516631, MyBioSource, San Diego, CA, USA; 1:1000), rabbit anti-EGFR (2232S, Cell Signaling Technology; 1:500), and rabbit anti-GAPDH (GTX100118, GeneTex, Irvine, CA, USA; 1:1000). After primary antibody incubation, the membranes were washed three times with TBS-T for 10 min each and then incubated with horseradish peroxidase (HRP)-conjugated secondary antibodies (1:5000; Cell Signaling Technology) diluted in 2% BSA for 1 h at room temperature. The protein bands were visualized using an enhanced chemiluminescence detection system (Clarity Western ECL kit, Bio-Rad Laboratories) and detected with a ChemiDoc XRS+ system (Bio-Rad Laboratories). Band intensities were quantified using ImageJ software (version 1.54s; Wayne Rasband, NIH, Bethesda, MD, USA), and relative protein expression was determined by normalization to GAPDH expression. No primary antibody controls were included to verify nonspecific binding, and all antibodies produced bands at their predicted molecular weights, confirming target specificity. Protein lysates for Western blotting were obtained from the same animal cohorts described above (Nor; *n* = 6, NTx; *n* = 8, Tx; *n* = 6).

### 2.7. Oil Red O Staining

Frozen ovarian tissues were sectioned at a thickness of 8 μm and fixed with 4% paraformaldehyde (PFA; Sigma-Aldrich, St. Louis, MO, USA) for 20 min at room temperature. After fixation, the sections were washed three times in 1× phosphate-buffered saline (PBS) for 3 min each and placed in a humidified chamber. To facilitate lipid staining, 100% propylene glycol (Polyethylene glycol 300; 6551-1400; Daejung, Siheung, Republic of Korea) was applied to the slides for 5 min and then removed; this step was repeated twice. The solution was then completely aspirated, and Oil Red O working solution (01391; Sigma-Aldrich, St. Louis, MO, USA) was added to the sections, which were subsequently incubated in a dry oven at 60 °C for 1 h. After being incubated, the slides were rinsed with 85% propylene glycol (180323-02; Electron Microscopy Sciences, Hatfield, PA, USA) for 5 min at room temperature and then washed three times with distilled water for 3 min each. Counterstaining was performed with Mayer’s hematoxylin (Dako, Carpinteria, CA, USA) for 1 min, followed by three additional washes in distilled water for 3 min each. Finally, the slides were mounted and examined under a bright-field microscope (Zeiss, Oberkochen, Germany). Lipid accumulation was quantified using ImageJ software (NIH, Bethesda, MD, USA). Ovaries used for Oil Red O analysis were obtained from the same experimental groups described in the TAA model section.

### 2.8. Hydroxyproline Assay

To measure total collagen content in ovarian tissues, a hydroxyproline assay (Hydroxyproline Assay Kit, MAK463, Sigma-Aldrich, St. Louis, MO, USA) was performed. The experiment was conducted according to the manufacturer’s instructions. Briefly, tissue samples were hydrolyzed by mixing with 10 N NaOH and incubating at 100 °C for 1 h, followed by neutralization with 10 N HCl. The samples were diluted with distilled water and centrifuged to remove debris. Standard solutions were prepared using the provided hydroxyproline standard. Samples and standards were then added to a 96-well plate and reacted with the reaction mixture containing Reagent A and Oxidation Buffer. After incubation at room temperature, Reagent B was added, and the plate was further incubated at 37 °C for 90 min. The optical density (OD) was measured at 560 nm using a microplate reader (BioTek Instruments). Hydroxyproline concentration was calculated based on the standard curve using the following equation: Hydroxyproline (μg/mL) = (OD_sample − OD_blank) × dilution factor/slope of the standard curve.

### 2.9. Immunohistochemical Staining

Paraffin-embedded ovarian tissues were cut into 4 µm sections and heated in a dry oven at 60 °C for 1 h. After the tissue had cooled sufficiently at room temperature, the sections were then deparaffinized with xylene and rehydrated through a graded ethanol series. Antigen retrieval was performed using EDTA-based buffer (eLbio, Seongnam-si, Republic of Korea), followed by rinsing under running tap water. Endogenous peroxidase activity was quenched with 3% hydrogen peroxide in methanol for 10 min at room temperature. After being washed, the sections were incubated overnight at 4 °C with primary antibody diluted in antibody diluent buffer (Dako, Carpinteria, CA, USA). For PlGF staining, a rabbit anti-PlGF antibody (bs-0280R; Bioss Antibodies; Woburn, MA, USA) was used at a 1:200 dilution. After being rinsed, the slides were incubated for 1 h at room temperature with Real EnVision HRP-conjugated secondary antibody (Dako, Carpinteria, CA, USA). Visualization was achieved by staining with 3,3′-diaminobenzidine (DAB; Dako), and the tissues were counterstained with Mayer’s hematoxylin (Dako) for 1 min. Following dehydration with ethanol and xylene, the slides were mounted and examined under a bright-field microscope (Zeiss, Oberkochen, Germany). Negative controls were prepared by omitting the primary antibody to confirm the absence of nonspecific HRP staining. The observed PlGF staining pattern was consistent with previously reported cellular localization, supporting antibody specificity. Staining intensity was quantified using ImageJ software by measuring the DAB-positive area across three non-overlapping fields per section. All IHC analyses were performed using the same animal cohorts described in the TAA model section.

### 2.10. Terminal Deoxynucleotidyl Transferase dUTP Nick End Labeling (TUNEL) Assay

Apoptotic cells in ovarian tissue were detected using a TUNEL assay kit (ab206386; Abcam, Waltham, MA, USA) according to the manufacturer’s instructions. Paraffin-embedded ovarian tissues were cut into 4 μm sections, incubated at 60 °C for 1 h, and deparaffinized with xylene and graded ethanol. After rehydration, the sections were permeabilized with proteinase K, and endogenous peroxidase activity was blocked with 3% hydrogen peroxide in methanol at room temperature. The tissues were equilibrated in TdT buffer and incubated with TdT enzyme and labeling mix in a humidified chamber. The labeling reaction was stopped using the provided stop buffer, followed by incubation with the conjugate solution. Signal detection was performed with 3,3′-diaminobenzidine (DAB), and counterstaining was carried out with methyl green. Finally, the slides were mounted with organic mounting medium. As a positive control, sections were treated with DNase I (1 µg/µL in TBS/1 mM MgSO_4_) for 20 min at room temperature to induce DNA fragmentation, whereas negative controls were processed without the enzyme TdT. All stained tissues were scanned using a digital slide scanner (3DHISTECH Ltd., Budapest, Hungary), and apoptotic signals were quantified using ImageJ software (NIH, Bethesda, MD, USA). All TUNEL analyses were performed using the same animal cohorts described in the TAA model section.

### 2.11. TMRE-Mitochondrial Membrane Potential Assay

To evaluate mitochondrial membrane potential in cells, a TMRE assay (TMRE Mitochondrial Membrane Potential Assay Kit, ab113852, Abcam, UK) was performed. The experiment was conducted according to the manufacturer’s instructions. Briefly, KGN cells were cultured and treated under the indicated experimental conditions. For depolarization control, KGN was treated with FCCP prior to staining. TMRE working solution was prepared in culture media and added to the cells, followed by incubation for 20 min under dark conditions. After incubation, cells were washed with PBS containing 0.2% BSA to remove excess dye. Fluorescence intensity was detected using a fluorescence microscope (EVOS M5000 Imaging System, Invitrogen, Carlsbad, CA, USA) at excitation/emission wavelengths of 549/575 nm. The relative mitochondrial membrane potential (ΔΨm) was expressed based on TMRE fluorescence intensity. Quantification of fluorescence intensity was performed using ImageJ software (NIH, Bethesda, MD, USA). The red channel was isolated from RGF images, and regions of interest (ROI) were defined for individual cells. Fluorescence intensity was measured for each cell and normalized per cell to account for differences in cell number among groups.

### 2.12. Immunofluorescence Staining

Ovarian tissues embedded in frozen blocks were cut into 8 μm thick sections and fixed in cold methanol for 20 min. After air drying, the sections were permeabilized with 0.25% Triton X-100 (T9284; Sigma-Aldrich, St. Louis, MO, USA) in phosphate-buffered saline (PBS) for 1 h at room temperature, followed by three washes with 1× PBS at room temperature for 5 min each. The excess PBS was removed, and the slides were placed in a humidified chamber. The tissue sections were then incubated with blocking solution (Dako, Carpinteria, CA, USA) for 1 h at room temperature, followed by overnight incubation at 4 °C with the following primary antibodies diluted in antibody buffer (S3022, Dako, Carpinteria, CA, USA): rabbit anti-Flt-1 (NBP3-11864, Novus Biologicals, Centennial, CO, USA; 1:200) and rabbit anti-phospho-Nrf2 (bs-2013R, Bioss, Woburn, MA, USA; 1:200). Negative control sections were treated with diluent buffer without primary antibody. After being washed three times with PBS for 5 min each, the samples were incubated with the appropriate secondary antibody for 1 h at room temperature. The sections were counterstained and mounted using DAPI-containing mounting medium (Vectashield, Burlingame, CA, USA). Stained tissues were visualized under a fluorescence microscope (Zeiss LSM 780, Oberkochen, Germany) at 40× and 63× magnification. Representative images were captured, and image processing was performed using ImageJ software (NIH, Bethesda, MD, USA). All IF analyses were performed using the same biological replicates described in the KGN experiment section.

### 2.13. MitoTracker and MitoSOX Staining

Frozen ovarian tissue blocks were cut into 8 μm slices and fixed with methanol for 20 min at room temperature. Cultured cells were also fixed with methanol under the same conditions. After fixation, tissue sections and cells were washed with 1× phosphate-buffered saline (PBS) and incubated with Hank’s balanced salt solution (HBSS) containing 1.5 μM MitoSOX (Invitrogen, Carlsbad, CA, USA) and 50 nM MitoTracker (Invitrogen, Carlsbad, CA, USA) for 40 min at 37 °C in a dry oven. After incubation, the samples were rinsed with 1× PBS. The stained ovarian tissues and cells were visualized using a fluorescence microscope, and representative images were captured. Image analysis was performed using ImageJ software (Wayne Rasband, National Institutes of Health, Bethesda, MD, USA). In vivo analyses were conducted using the same animal cohorts described in the TAA model section, whereas in vitro staining was performed using three independent biological replicates (*n* = 3).

### 2.14. Hematoxylin and Eosin Staining for Follicle Counting

Ovarian tissues were fixed in 10% neutral buffered formalin (NBF; BBC Biochemical, Washington, DC, USA) and processed for paraffin embedding. Paraffin blocks were cut at a thickness of 4 μm, and the sections were deparaffinized in a dry oven at 60 °C, followed by sequential immersion in xylene and graded ethanol. After being rinsed in tap water, the sections were stained with Harris hematoxylin (Leica Biosystems, Wetzlar, Germany) for 7 min, briefly dipped in 0.1% HCl for 2 s, and counterstained with alcoholic eosin Y (Sigma-Aldrich, St. Louis, MO, USA). The stained slides were scanned using a digital slide scanner (3D HISTECH Ltd., Budapest, Hungary) to capture the entire ovarian tissue. For follicle quantification, serial sections were cut at a thickness of 4 μm, and counting was performed on every other section (i.e., one section was analyzed, and the next was discarded) of the ovary to avoid counting the same follicle twice. Follicles were categorized into primordial, primary, secondary, antral, and atretic stages on the basis of the classification system described in a previous report [29]. Counting was performed independently by at least three investigators to ensure reproducibility. All follicle counting analyses were performed using the same animal cohorts described above.

### 2.15. Picrosirius Red Staining

Ovarian tissues were fixed in 10% NBF (BBC Biochemical, Washington, DC, USA), embedded in paraffin, and cut into 4 μm thick sections. The sections were first heated in a dry oven at 60 °C for 1 h and cooled to room temperature, followed by deparaffinization using xylene and a graded ethanol series. A Picrosirius Red staining kit (Abcam, ab150681, Cambridge, UK) was subsequently used according to the manufacturer’s protocol. Briefly, the sections were rehydrated in distilled water for 2 min, incubated in Picrosirius Red solution for 60 min at room temperature, and rinsed with an acetic acid solution. The slides were then washed with absolute ethanol, dehydrated twice in absolute ethanol for 3 min each, and mounted. Stained sections were scanned using a digital slide scanner (3DHISTECH Ltd., Budapest, Hungary), and collagen deposition was quantified using ImageJ software (NIH, Bethesda, MD, USA). For quantitative analysis, three non-overlapping regions per ovary were selected at ×10 magnification, avoiding areas of tissue folding or section artifacts. Images were first converted to 16-bit grayscale, and a color threshold was applied using the Hue/Saturation/Brightness (HSB) mode to isolate birefringent collagen fibers. Threshold values were determined empirically using the TAA-treated group to ensure adequate detection of collagen-rich areas, and the same threshold range was uniformly applied to all samples across groups. Quantification was restricted to the ovarian stromal compartment by manually excluding follicles and corpora lutea. The fibrotic area was calculated as the percentage of PSR-positive pixels relative to the total stromal area. For quantitative PSR analysis, ovarian sections from Nor (*n* = 6), NTx (*n* = 8), and Tx (*n* = 6) rats were examined.

### 2.16. Enzyme-Linked Immunosorbent Assay (ELISA)

Blood samples were collected from the aortas of the rats in each experimental group at the time of sacrifice. Serum was separated using blood collection tubes (Vacutainer; BD Biosciences, San Jose, CA, USA) and stored at −80 °C until analysis. Hormone and metabolic factor concentrations were determined using the following ELISA kits according to the manufacturers’ protocols: rat AMH (CSB-E11162r, CUSABIO, Houston, TX, USA), rat estradiol (E2; MBS2607338, MyBioSource, San Diego, CA, USA), rat luteinizing hormone (LH; MBS764675, MyBioSource, San Diego, CA, USA), rat follicle-stimulating hormone (FSH; MBS720155, San Diego, CA, USA), rat testosterone (TES; MBS282195, MyBioSource, San Diego, CA, USA), rat soluble Flt-1 (sFlt-1; MBS007319, MyBioSource, San Diego, CA, USA) and human PlGF (DPG00, R&D Systems, Minneapolis, MN, USA). Briefly, equal volumes of serum samples were loaded into antibody-coated wells, followed by incubation with horseradish peroxidase (HRP)-conjugated detection antibodies. After the substrate solution was added and allowed to develop in the dark, the absorbance was measured at the appropriate wavelength using a microplate reader (BioTek Instruments, Winooski, VT, USA). Concentrations of the target factors were calculated on the basis of standard curves generated for each factor. Serum ELISA analysis was performed on the same animal cohorts described in the TAA model section, whereas supernatant ELISA analysis was conducted using three independent biological replicates (*n* = 3).

### 2.17. Blood Chemistry Test

Serum levels of high-density lipoprotein (HDL) and low-density lipoprotein (LDL), the homeostatic model assessment of insulin resistance (HOMA-IR) index and homeostatic model assessment of insulin sensitivity (HOMA-IS) index, and total cholesterol levels were measured at the Southeast Medi-Chem Institute (Busan, Republic of Korea). Blood chemistry analyses were performed using the same animals described in the TAA model section.

### 2.18. Statistical Analysis

All experiments were performed in duplicate or triplicate under identical conditions, with independent biological replicates where applicable. Data are presented as mean ± standard error of the mean (SEM). Statistical analyses were conducted using R statistical software (version 1.8.25; R Foundation for Statistical Computing, Vienna, Austria). Normality of data distribution was assessed prior to analysis. Because the data did not consistently meet the assumptions of normality, non-parametric tests were used. Comparisons among multiple groups were performed using the Kruskal–Wallis test, followed by post hoc pairwise comparisons using Conover’s test with Benjamini–Hochberg correction for multiple testing. Differences were considered statistically significant at *p* < 0.05.

## 3. Results

### 3.1. PD-MSC Transplantation Improves Metabolic and Endocrine Alterations in TAA-Induced Ovarian Injury

**Figure 1:**
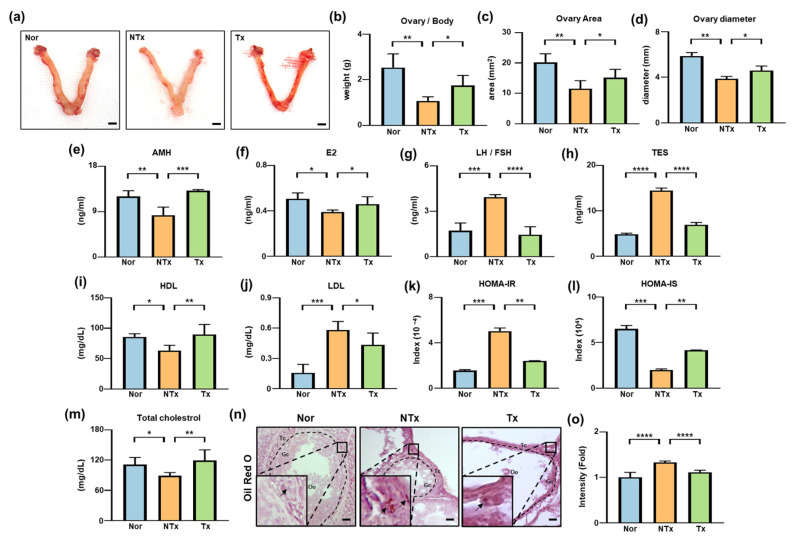
Effects of PD-MSCs on TAA-induced ovarian metabolic disorder. (**a**) Representative images of whole ovaries. Scale bar = 1 mm. (**b**–**d**) Ovary-to-body weight ratio, ovary area, and ovary diameter. (**e**–**h**) Serum hormone levels: anti-Müllerian hormone (AMH), estradiol (E2), luteinizing hormone (LH)/follicle-stimulating hormone (FSH) ratio, and testosterone (TES). (**i**–**m**) Serum metabolic parameters: high-density lipoprotein (HDL), low-density lipoprotein (LDL), HOMA-IR, HOMA-IS, and total cholesterol. (**n**) Oil Red O staining of ovarian tissue. Dotted lines indicate the boundary of follicles, and black arrows indicate Oil Red O-positive lipid accumulation regions within the ovarian tissue. Oo, oocyte; Gc, granulosa cell; Tc, theca cell. Scale bar = 50 μm. (**o**) Quantification of Oil Red O staining intensity using ImageJ. The rats were assigned to three groups: the Nor (control), NTx (TAA-treated), and Tx (TAA-induced injury and PD-MSC transplantation) groups. Data are presented as the mean ± SEM and were analyzed by the Kruskal–Wallis test followed by Conover’s post hoc test with the Benjamini–Hochberg correction. * *p* < 0.05, ** *p* < 0.01, *** *p* < 0.001, **** *p* < 0.0001.

TAA administration induces systemic metabolic dysfunction and endocrine imbalance [30]. To determine whether PD-MSC transplantation attenuates these effects, we evaluated ovarian morphology, serum hormone levels, and metabolic parameters (Figure 1).

Ovarian morphology analysis showed that the ovaries in the TAA-treated group were significantly smaller than those in the control group, whereas ovarian size was partially restored following PD-MSC transplantation (Figure 1a). The ovary-to-body weight ratio, ovarian area, and ovarian diameter were significantly reduced in the TAA-treated group compared with the control group (** *p* < 0.01; Figure 1b–d) but were significantly increased after PD-MSC transplantation (* *p* < 0.05).

Serum concentrations of anti-Müllerian hormone (AMH) and estradiol (E2), markers of ovarian reserve, were significantly lower in the TAA-treated group than in the control group (** *p* < 0.01), whereas their levels were significantly increased in the PD-MSC transplantation group (* *p* < 0.05; Figure 1e,f). In contrast, the luteinizing hormone (LH)-to-follicle-stimulating hormone (FSH) ratio and testosterone (TES) level were significantly higher in the TAA-treated group than in the control group (*** *p* < 0.001), but these increases were significantly reduced after PD-MSC transplantation (*** *p* < 0.001; Figure 1g,h).

TAA also induces metabolic alterations, particularly lipid metabolism. Serum analysis revealed that high-density lipoprotein (HDL) was significantly reduced in the TAA-treated group (* *p* < 0.05) but increased in the PD-MSC transplantation (** *p* < 0.01; Figure 1i). Conversely, low-density lipoprotein (LDL) levels were significantly higher in the TAA-treated group than in the control group (*** *p* < 0.001) and were significantly reduced after PD-MSC transplantation (* *p* < 0.05; Figure 1j). Homeostatic model assessment indices further supported these findings. HOMA-IR was significantly elevated in the TAA-treated group (*** *p* < 0.001) and significantly reduced following PD-MSC transplantation (** *p* < 0.01), whereas HOMA-IS was significantly decreased in the TAA-treated group (*** *p* < 0.001) and increased after PD-MSC transplantation (** *p* < 0.01; Figure 1k,l). Total cholesterol levels were significantly lower in the TAA-treated group than in the control group (* *p* < 0.05) and significantly increased following PD-MSC transplantation (** *p* < 0.01; Figure 1m). These alterations were consistent with increased lipid accumulation in ovarian tissues from the TAA-treated group, as confirmed by Oil Red O staining. Lipid accumulation was significantly reduced in the PD-MSC transplantation group (**** *p* < 0.0001; Figure 1n,o).

Together, these results indicate that PD-MSC transplantation attenuates metabolic and endocrine alterations associated with TAA-induced ovarian injury.

### 3.2. PD-MSC Transplantation Attenuates Fibrosis via Regulation of TGF-β1/SMAD Signaling in TAA-Injured Ovaries

**Figure 2:**
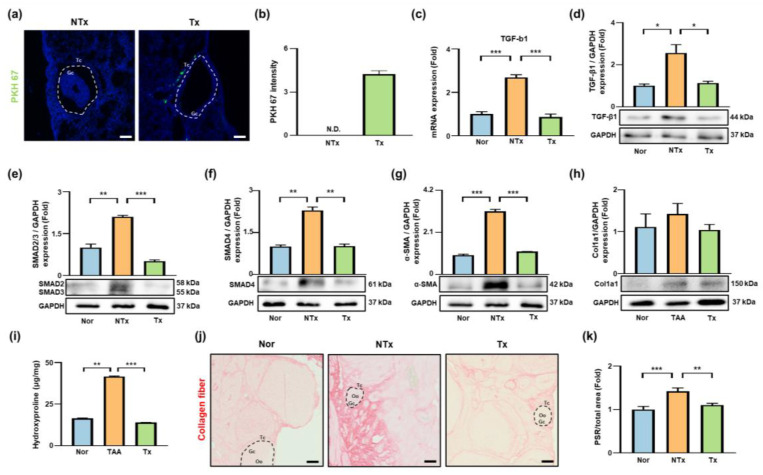
PD-MSCs exert antifibrotic effects by suppressing TGF-β1/SMAD signaling. (**a**) Localization of PKH 67-labeled PD-MSCs in ovarian tissues. White dashed lines indicate the boundary of follicles. PKH-positive cells (green) indicate transplanted cells; nuclei are stained with DAPI (blue). Scale bar = 50 µm. (**b**) Quantification of PKH67 fluorescence intensity. (**c**) TGF-β1 mRNA expression. (**d**–**h**) Protein expression of TGF-β1, phosphorylated SMAD 2/3, SMAD 4, α-SMA, and Col1a1. (**i**) Total collagen content measured by hydroxyproline assay. (**j**) Picrosirius Red (PSR) staining of collagen fibers in ovarian tissue. Black dashed lines indicate the boundary of follicles. Oo, oocyte; Gc, granulosa cell; Tc, theca cell. Scale bar = 50 μm. (**k**) Quantification of the PSR-positive area. The rats were assigned to three groups: the Nor (control), NTx (TAA-injured), and Tx (TAA-induced injury and PD-MSC transplantation) groups. Data are presented as the mean ± SEM and were analyzed by the Kruskal–Wallis test followed by Conover’s post hoc test with the Benjamini–Hochberg correction. * *p* < 0.05, ** *p* < 0.01, *** *p* < 0.001.

Transforming growth factor-β (TGF-β) signaling is a key regulator of fibrosis and ovarian dysfunction, promoting ECM deposition and the expression of fibrosis-associated markers such as α-smooth muscle actin (α-SMA) and collagen type I alpha 1 (Col1a1) via downstream SMAD transcription factors [31]. To determine whether PD-MSC transplantation modulates this pathway, we evaluated TGF-β/SMAD signaling activity and fibrosis-related markers (Figure 2).

PKH 67 fluorescence signals were detected in ovarian tissues and were primarily localized around the theca cell layer adjacent to blood vessels in the PD-MSC transplantation group, indicating successful engraftment following intravenous transplantation (Figure 2a,b).

TGF-β1 mRNA expression was significantly increased in the TAA-treated group compared with the control group (*** *p* < 0.001) and was significantly reduced following PD-MSC transplantation (*** *p* < 0.001; Figure 2c). Similarly, TGF-β1 protein levels were elevated in the TAA-treated group (* *p* < 0.05) and decreased after PD-MSC transplantation (* *p* < 0.05; Figure 2d). SMAD2/3 and SMAD4 protein expression showed a similar pattern, with significant increases in the TAA-treated group (** *p* < 0.01) and significant reductions following PD-MSC transplantation (** *p* < 0.01, *** *p* < 0.001; Figure 2e,f).

The expression of fibrosis markers was also affected. α-SMA mRNA and protein levels were significantly elevated in the TAA-treated group (** *p* < 0.01) and significantly reduced following PD-MSC transplantation (*** *p* < 0.001; Figure 2g, Supplementary Figure S1a). Similarly, Col1a1 mRNA expression was significantly higher in the TAA-treated group than in the control group (*** *p* < 0.001) and decreased after PD-MSC transplantation (*** *p* < 0.001; Supplementary Figure S1b). Consistent with these findings, Col1a1 protein expression was increased in the TAA-treated group and reduced in the PD-MSC transplantation group (Figure 2h).

Total collagen content, assessed by hydroxyproline assay, was significantly elevated in the TAA-treated group (** *p* < 0.01) and significantly reduced following PD-MSC transplantation (*** *p* < 0.001; Figure 2i). Picrosirius red (PSR) staining further confirmed increased collagen deposition in TAA-treated ovaries (*** *p* < 0.001), which was significantly reduced after PD-MSC transplantation (** *p* < 0.01; Figure 2j,k).

Collectively, these results indicate that PD-MSC transplantation attenuates fibrosis in TAA-treated ovaries, accompanied by reducing collagen accumulation and downregulating of fibrosis-related markers.

### 3.3. PD-MSC Transplantation Is Associated with Activation of PlGF/Flt-1-Related Signaling in TAA-Injured Ovaries

**Figure 3:**
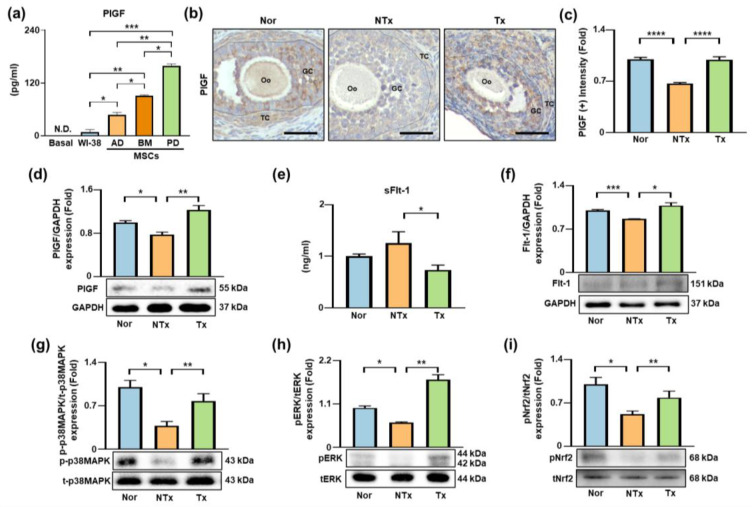
Effects of PD-MSCs on the PlGF signaling pathway in TAA-injured ovaries. (**a**) PlGF levels in ocnditioned media from WI-38, AD-MSCs, BM-MSCs, and PD-MSCs. N.D., not detected. (**b**) Localization of PlGF in ovarian tissue. (**c**) Quantification of PlGF expression. Oo, oocyte; GC, granulosa cell; TC, theca cell. Scale bar = 50 μm. (**d**) PlGF protein expression. (**e**) Serum sFlt-1 levels. (**f**) Flt-1 protein expression. (**g**–**i**) Phosphorylated (p-) and total (t-) p38 MAPK, ERK, and Nrf2 protein expression. The rats were divided into three groups: the Nor (control), NTx (TAA-injured), and Tx (TAA-induced injury and PD-MSC transplantation) groups. Data are presented as the mean ± SEM and were analyzed by the Kruskal–Wallis test followed by Conover’s post hoc test with Benjamini–Hochberg correction. * *p* < 0.05, ** *p* < 0.01, *** *p* < 0.001, **** *p* < 0.0001.

PlGF regulates angiogenesis, inflammation, oxidative stress, and tissue homeostasis under stress conditions [17]. To determine whether PD-MSC transplantation alters PlGF/Flt-1 signaling in TAA-treated ovaries, we evaluated PlGF secretion, localization, and Flt-1 receptor dynamics, along with its related pathway (Figure 3).

ELISA analysis showed that PD-MSCs secreted significantly higher levels of PlGF than other cell types, including WI-38 fibroblasts (18-fold, *** *p* < 0.001), AD-MSCs (3-fold, ** *p* < 0.01), and BM-MSCs (approximately 2-fold, * *p* < 0.05; Figure 3a).

Immunohistochemical analysis revealed that PlGF was predominantly localized in granulosa cells within ovarian follicles. PlGF expression was significantly decreased in the TAA-treated group (**** *p* < 0.0001) and increased following PD-MSC transplantation (**** *p* < 0.0001; Figure 3b,c). Western blot analyses showed a similar pattern, with reduced PlGF protein levels in the TAA-treated group (* *p* < 0.05) and significantly increased levels after PD-MSC transplantation (** *p* < 0.01).

To evaluate receptor dynamics, levels of soluble Flt-1 (sFlt-1), a decoy receptor for PlGF, were measured. sFlt-1 levels increased more in the TAA-treated group than in the control group and significantly reduced following PD-MSC transplantation (* *p* < 0.05; Figure 3e). In contrast, the membrane-bound Flt-1 protein expression was significantly downregulated in the TAA-treated group (*** *p* < 0.001), but its expression was significantly restored after PD-MSC transplantation (* *p* < 0.05; Figure 3f). Notably, phosphorylated p38 MAPK and ERK levels were significantly decreased in the TAA-treated group (* *p* < 0.05) but increased following PD-MSC transplantation (** *p* < 0.01; Figure 3g,h). Nrf2 activation, assessed as the ratio of phosphorylated to total Nrf2, was significantly decreased in the TAA-treated group (* *p* < 0.05) and was significantly increased after PD-MSC transplantation (** *p* < 0.01; Figure 3i).

Collectively, these findings suggest that PD-MSC transplantation is associated with changes in PlGF/Flt-1-related signaling components and downstream pathway activity associated with antioxidant responses in TAA-injured ovaries.

### 3.4. Antioxidant Effects of PD-MSC Transplantation in TAA-Injured Ovaries

**Figure 4:**
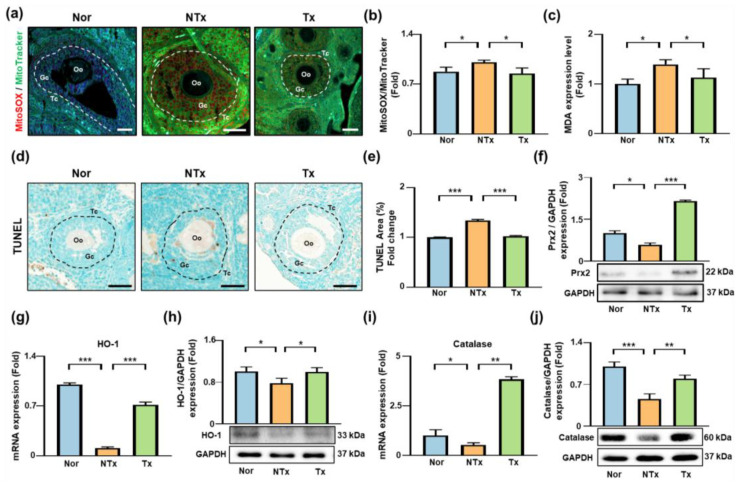
Effects of PD-MSCs on antioxidant activity in TAA-injured ovaries. (**a**) MitoSOX and MitoTracker staining in ovarian tissues. Oo, oocyte; Gc, granulosa cell; Tc, theca cell. Scale bar = 50 μm. Dashed circles indicate representative follicular regions. (**b**) Quantification of MitoSOX and MitoTracker fluorescence intensity. (**c**) Malondialdehyde (MDA) levels. (**d**) TUNEL staining of apoptotic cells in ovarian tissue. Scale bar = 50 μm. Dashed circles indicate representative follicular regions. (**e**) Quantification of apoptotic cells. (**f**–**j**) Expression of antioxidant markers: Prx2 protein, HO-1 mRNA and protein, and Catalase mRNA and protein. The rats were divided into three groups: the Nor (control), NTx (TAA-injured), and Tx (TAA-induced injury and PD-MSC transplantation) groups. Data are presented as the mean ± SEM and were analyzed by the Kruskal–Wallis test followed by Conover’s post hoc test with the Benjamini–Hochberg correction. * *p* < 0.05, ** *p* < 0.01, *** *p* < 0.001.

TAA administration induces oxidative stress by increasing ROS levels and promoting lipid peroxidation, leading to cellular injury [32]. To determine whether PD-MSC transplantation modulates these effects, we evaluated mitochondrial ROS levels, lipid peroxidation, apoptosis, and antioxidant enzyme expression in ovarian tissues (Figure 4).

MitoSOX/MitoTracker staining showed that mitochondrial ROS levels were significantly increased in the TAA-treated group compared with the control group (* *p* < 0.05) but were significantly reduced following PD-MSC transplantation (* *p* < 0.05; Figure 4a,b). Consistent with these findings, malondialdehyde (MDA) levels, an indicator of lipid peroxidation, were significantly elevated in the TAA-treated group (* *p* < 0.05) and significantly decreased after PD-MSC transplantation (* *p* < 0.05; Figure 4c). Analysis of oxidative damage-induced apoptosis using TUNEL staining revealed a significant increase in apoptotic cells in the TAA-treated group (*** *p* < 0.001), which was significantly reduced following PD-MSC transplantation (*** *p* < 0.001; Figure 4d,e).

Expression of antioxidant enzymes was also affected. Prx2 protein levels were significantly decreased in the TAA-treated group (* *p* < 0.05) and significantly increased after PD-MSC transplantation (**** *p* < 0.0001; Figure 4f). Heme oxygenase-1 (HO-1) mRNA expression was significantly decreased in the TAA-treated group (*** *p* < 0.001) and significantly increased following PD-MSC transplantation (*** *p* < 0.001; Figure 4g), with a similar pattern observed at the protein level (Figure 4h). Catalase mRNA and protein expression were significantly decreased in the TAA-treated group (* *p* < 0.05, *** *p* < 0.001) and significantly increased following PD-MSC transplantation (** *p* < 0.01; Figure 4i,j).

Together, these results indicate that PD-MSC transplantation attenuates oxidative stress in TAA-treated ovaries, accompanied by reduced ROS production, lipid peroxidation, and apoptosis, as well as increased expression of antioxidant enzymes.

### 3.5. Changes in Ovarian Architecture and Folliculogenesis After PD-MSC Transplantation

**Figure 5:**
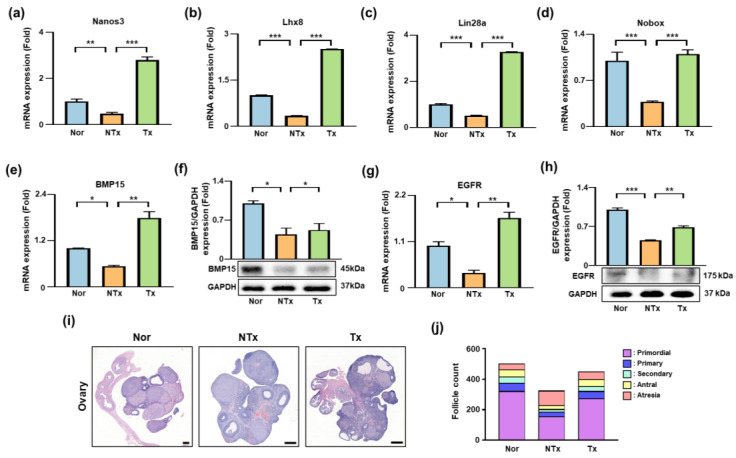
Effects of PD-MSCs on ovarian architecture and folliculogenesis (**a**–**e**) The mRNA expression of folliculogenesis-related genes: *Nanos3*, *Lhx8*, *Lin28a*, *Nobox*, and *BMP15*. (**f**,**h**) Protein expression of BMP15 and EGFR. (**g**) *EGFR* mRNA expression. (**i**) Representative H&E-stained ovarian sections showing follicle morphology and distribution. Scale bar = 500 μm. (**j**) Quantification of follicle distribution at different developmental stages. The rats were divided into three groups: the Nor (control), NTx (TAA-injured), and Tx (TAA-induced injury and PD-MSC transplantation) groups. Data are presented as the mean ± SEM and were analyzed by the Kruskal–Wallis test followed by Conover’s post hoc test with the Benjamini–Hochberg correction. * *p* < 0.05, ** *p* < 0.01, *** *p* < 0.001.

Folliculogenesis is a tightly regulated process involving multiple transcription factors and growth regulators that control the progression of germ cells through distinct follicular stages. Disruption of follicle development impairs endocrine function, reduces oocyte quality, and contributes to ovarian dysfunction [33]. To determine whether PD-MSC transplantation affects TAA-induced alterations in follicular development, we evaluated the expression of folliculogenesis-related genes and performed histological analysis of ovarian tissues (Figure 5).

Early germ cell-associated genes, including *Nanos3*, *Lhx8*, and *Lin28a*, are essential for the differentiation of primordial germ cells into primordial follicles [26]. The mRNA expression was significantly decreased in the TAA-treated group compared with the control group (** *p* < 0.01) and significantly increased after PD-MSC transplantation (**** *p* < 0.0001; Figure 5a–c). *Nobox* expression, which regulates the transition from primordial to primary follicles, showed a similar pattern, with reduced levels in the TAA-treated group (*** *p* < 0.001) and significantly increased after PD-MSC transplantation (*** *p* < 0.001; Figure 5d).

Expression of BMP15, a key regulator of primary follicle growth and oocyte–granulosa cell communication, was decreased in the TAA-injured group (* *p* < 0.05) and significantly increased following PD-MSC transplantation at both mRNA and protein levels (** *p* < 0.01, * *p* < 0.05; Figure 5e,f). EGFR expression, which is involved in antral follicle maturation, also showed reduced mRNA and protein levels in the TAA-treated group (* *p* < 0.05, *** *p* < 0.001) and increased levels after PD-MSC transplantation (** *p* < 0.01; Figure 5g,h).

Histological analysis supported these findings. Ovaries from the TAA-treated group exhibited reduced follicle numbers and altered distribution of follicle stages, whereas ovaries from the PD-MSC transplantation group showed increased numbers of developing follicles (Figure 5i). Quantitative analysis revealed that primordial, primary, secondary, and antral follicle counts were significantly lower in the TAA-treated group than in the control group, while PD-MSC transplantation increased follicle numbers across multiple developmental stages and reduced the proportion of atretic follicles (Figure 5j; Table 1).

Overall, these results indicate that PD-MSC transplantation is associated with increased expression of folliculogenesis-related genes and proteins, preservation of ovarian architecture, and changes in follicle distribution in TAA-treated ovaries.

### 3.6. PD-MSCs Coculture Is Associated with Modulation of PlGF/Flt-1-Related Signaling and Reduces Oxidative Stress in KGN Cells

**Figure 6:**
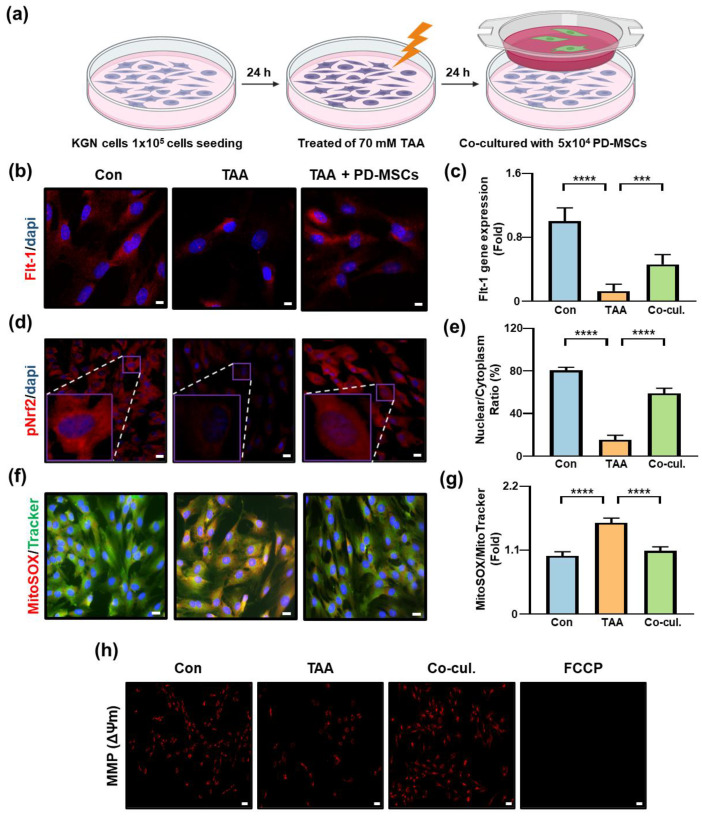
The antioxidant effects of PD-MSCs on KGN cells are mediated by the activation of PlGF signaling. (**a**) Schematic diagram showing the experimental workflow. (**b**,**d**) Immunofluorescence staining of Flt-1 and phosphorylated Nrf2 (pNrf2) in KGN cells. Nuclei are stained with DAPI. Dash boxes indicate magnified reigions. Scale bar = 20 μm. (**c**,**e**) Quantification of Flt-1 fluorescence intensity and pNrf2 nuclear-to-cytoplasmic ratio. (**f**) MitoSOX and MitoTracker staining. Green fluorescence indicates MitoTracker staining, and red fluorescence indicates MitoSOX staining. Scale bar = 20 μm. (**g**) Quantification of MitoSOX and MitoTracker fluorescence intensity. (**h**) Mitochondrial membrane potential assessed by TMRE staining. Scale bar = 100 μm. Cells were assigned to three groups: Con, Control; TAA, TAA-treated; Co-cul., TAA-treated and cocultured with PD-MSCs, and FCCP-treated (positive control for mitochondrial depolarization). Data are presented as the mean ± SEM and were analyzed by the Kruskal–Wallis test followed by Conover’s post hoc test with the Benjamini–Hochberg correction. *** *p* < 0.001, **** *p* < 0.0001.

PlGF signaling has been implicated in oxidative stress regulation, but its direct effects on ovarian granulosa cells remain insufficiently explored [34,35]. To examine whether PD-MSC coculture is associated with activation of PlGF-related downstream signaling in ovarian cells, we established an in vitro coculture system using TAA-treated KGN cells (Figure 6a).

Immunofluorescence analysis showed that Flt-1 expression was significantly reduced in TAA-treated KGN cells compared with control cells (**** *p* < 0.0001) and significantly increased following coculture with PD-MSCs (**** *p* < 0.0001; Figure 6b,c).

Nrf2 activation was also evaluated. Nuclear translocation of phosphorylated Nrf2 (pNrf2) was substantially reduced in the TAA-treated group but restored after PD-MSC coculture (Figure 6d). Quantitative analysis of the Nrf2 nuclear-to-cytoplasmic ratio confirmed increased nuclear accumulation of pNrf2 in the coculture group compared with the TAA-treated group (**** *p* < 0.0001; Figure 6e).

Mitochondrial ROS levels were assessed using MitoSOX/MitoTracker staining. TAA treatment significantly increased mitochondrial ROS production (**** *p* < 0.0001), whereas PD-MSC coculture significantly reduced ROS levels (**** *p* < 0.0001; Figure 6f,g).

In addition, mitochondrial membrane potential (ΔΨm) was evaluated using TMRE staining. TMRE fluorescence intensity was significantly reduced in the TAA group (*** *p* < 0.001), indicating loss of mitochondrial membrane potential, and increased following PD-MSC coculture (**** *p* < 0.0001; Figure 6h, Supplementary Figure S2b).

Collectively, these results suggest that PD-MSC coculture is associated with activation of Flt-1 signaling, enhanced Nrf2 nuclear translocation, reduced mitochondrial ROS production, and stabilization of mitochondrial membrane potential in KGN cells.

### 3.7. Effects of PlGF Knockdown on PD-MSC-Mediated Antioxidant Responses

**Figure 7:**
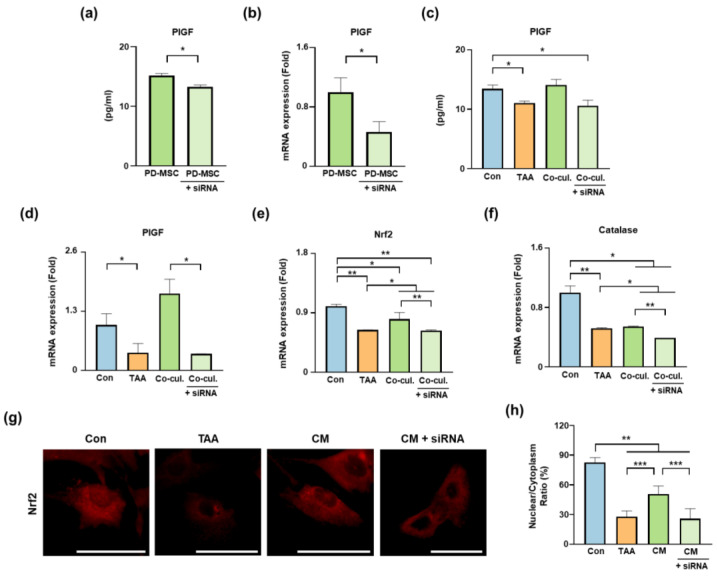
PlGF knockdown in PD-MSCs attenuates coculture-mediated antioxidant signaling. (**a**,**b**) Validation of PlGF knockdown in PD-MSCs by ELISA and qRT-PCR. (**c**,**d**) PlGF levels in KGN cell supernatants and mRNA expression under coculture conditions. (**e**,**f**) Relative mRNA expression of *Nrf2* and *Catalase* in KGN cells. (**g**) Immunofluorescence analysis of Nrf2 localization. Scale bar = 50 μm. (**h**) Quantification of nuclear-to-cytoplasmic ratio of Nrf2. For coculture experiments (**c**–**f**), KGN cells were assigned to four groups: control (Con), TAA-treated (TAA), TAA-treated coculture with PD-MSCs (Co-cul.), and TAA-treated cocultured with siPlGF-transfected PD-MSCs (Co-cul. + siRNA). For conditioned media (CM) experiments (**g**,**h**), KGN cells were treated with conditioned media derived from control PD-MSCs (CM) or siPlGF-transfected PD-MSCs (CM + siRNA) following TAA treatment. Data are presented as the mean ± SEM and were analyzed using the Kruskal–Wallis test followed by Conover’s post hoc test with Benjamini–Hochberg correction. * *p* < 0.05, ** *p* < 0.01, *** *p* < 0.001.

To evaluate the contribution of PD-MSC-derived PlGF to downstream signaling, PlGF expression was suppressed in PD-MSCs using siRNA. The efficiency of knockdown was confirmed by ELISA and qRT-PCR. PlGF levels in conditioned media from siPlGF-transfected PD-MSCs were significantly reduced compared with those from control PD-MSCs (* *p* < 0.05; Figure 7a), and PlGF mRNA expression was similarly decreased (* *p* < 0.05; Figure 7b).

Under coculture conditions, PlGF availability was assessed in KGN cell supernatants. TAA treatment significantly reduced PlGF levels compared with the control group (* *p* < 0.05; Figure 7c). Coculture with PD-MSCs increased PlGF levels, whereas this increase was attenuated when PD-MSCs were transfected with siPlGF (* *p* < 0.05). A similar pattern was observed at the mRNA level, with increased PlGF expression in the coculture group and reduced expression in the siPlGF condition (* *p* < 0.05; Figure 7d).

We next examined antioxidant-related gene expression. *Nrf2* and *Catalase* mRNA levels were increased in the PD-MSC cocultured group compared with the TAA group, whereas this induction was attenuated under siPlGF conditions (Figure 7e,f). Although the magnitude of change varied among targets, the overall trend was consistent, indicating that PlGF knockdown partially impairs PD-MSC-mediated antioxidant gene induction.

To further examine downstream signaling, KGN cells were treated with conditioned media (CM) derived from control or siPlGF-transfected PD-MSCs. Consistent trends were observed at the protein level, including Flt-1, expression and phosphorylation of p38 MAPK and ERK, which were increased in the PD-MSC CM group and reduced in the siPlGF CM condition (Supplementary Figure S3a).

Immunofluorescence analysis demonstrated increased nuclear localization of Nrf2 in the PD-MSC CM group, whereas this effect was attenuated following PlGF knockdown (Figure 7g). Quantitative analysis of the nuclear-to-cytoplasmic ratio confirmed reduced nuclear accumulation of Nrf2 in the siPlGF condition compared with the PD-MSC CM group (Figure 7h).

Collectively, these findings indicate that suppression of PD-MSC-derived PlGF attenuates activation of the Flt-1/p38 MAPK/ERK/Nrf2 signaling axis and partially attenuates associated antioxidant responses in KGN cells.

## 4. Discussion

In this study, PD-MSC transplantation was associated with marked improvement in ovarian architecture and redox status in a TAA-induced ovarian dysfunction model. These effects were accompanied by alterations in systemic metabolic parameters, endocrine balance, and follicular integrity, along with oxidative stress and stromal remodeling. These findings support the link between metabolic disturbance and ovarian dysfunction and suggest that PD-MSCs may exert coordinated effects at both systemic and local levels. Consistent with previous reports, MSC-based therapies have been shown to regulate endocrine function and reduce oxidative damage in ovarian injury models [36,37].

In addition, MSCs have been reported to support folliculogenesis and maintain the ovarian follicle pool [37]. However, most prior studies have described these effects at a general paracrine or antioxidant level, without identifying specific upstream mediators or signaling pathways [26,27]. In this context, our findings suggest that PD-MSC transplantation is associated with changes in redox balance and follicular dynamics in conjunction with activation of PlGF/Flt-1 signaling.

The present study identifies PlGF/Flt-1 signaling as a pathway associated with antioxidant and endocrine-related responses following PD-MSC transplantation. Although the paracrine effects of MSCs have been widely described [38], direct evidence linking PlGF-driven activation of the Flt-1/p38 MAPK/ERK/Nrf2 axis to ovarian injury models has been limited. Our findings suggest that activation of this pathway is associated with cytoprotective and redox-regulatory responses, rather than representing a single dominant mediator [39].

Furthermore, comparative analysis of different MSC types revealed that PD-MSCs exhibited higher levels of PlGF secretion, supporting their potential therapeutic advantage among MSC sources. Beyond mechanistic consideration, PD-MSCs possess intrinsic advantages as a therapeutic cell source. While MSCs are generally characterized by low immunogenicity and broad paracrine activity [40], PD-MSCs originate from perinatal tissues and retain a biologically youthful phenotype, exhibiting lower baseline senescence, higher proliferative capacity, and a more consistent trophic secretome compared with AD-MSCs or BM-MSCs [41]. In contrast, adult-derived MSCs are more susceptible to donor variability and age-related functional decline [42,43]. These properties may contribute to the functional stability of PD-MSCs under fibrotic and oxidative conditions and support their capacity to maintain trophic signaling, including PlGF-associated pathways [44].

Integrating our in vivo and in vitro findings with previous reports, PD-MSC-derived PlGF is associated with activation of Flt-1 and downstream p38 MAPK and ERK signaling. These changes coincided with increased Nrf2 phosphorylation and nuclear translocation, along with upregulation of antioxidant enzymes such as Prx2, HO-1, and Catalase. These molecular alterations were observed together with reduced oxidative stress markers, supporting an association between PlGF/Flt-1 signaling and antioxidant responses. This antioxidant environment may contribute to reduced cellular oxidative damage, improved granulosa cell viability, and enhanced follicular development.

In addition to its redox-related effects, PlGF has been implicated in angiogenesis, vascular remodeling, and tissue oxygen delivery, processes that are important for follicular growth and endocrine regulation [39,45]. Collectively, these findings provide a framework linking PD-MSC-derived PlGF signaling to changes in ovarian structure and redox homeostasis under conditions of metabolic and oxidative stress.

Suppression of PlGF expression in PD-MSCs using siRNA reduced the induction of Nrf2 activation in cocultured KGN cells, as evidenced by decreased nuclear localization of Nrf2 and reduced expression of antioxidant-related genes. These findings suggest a functional contribution of PD-MSC-derived PlGF to antioxidant signaling.

Consistently, PlGF knockdown attenuated the induction of Nrf2 and Catalase mRNA expression, although the magnitude of change varied among targets. This partial effect suggests that PlGF contributes to, but in not solely responsible for, PD-MSC-mediated redox regulation. These findings further support the notion that multiple PD-MSC-derived paracrine factors may act in concert to regulate antioxidant responses under oxidative stress conditions [46].

These observations suggest that improved redox regulation may be associated with changes in mitochondrial membrane potential, potentially contributing to granulosa cell viability and ovarian tissue homeostasis.

The biological effects of PlGF are highly context-dependent and vary across tissues and disease states. For example, reduced PlGF levels are a feature of preeclampsia, a condition associated with oxidative stress and impaired antioxidant defenses [47]. In contrast, inhibition of PlGF has been reported to enhance antioxidant responses in certain conditions, such as diabetic retinopathy [48]. These observations indicate that PlGF exerts tissue- and context-specific effects depending on the local microenvironment and signaling context.

In the ovary, where oxidative stress and metabolic imbalance are prominent features, our findings support an association between PlGF signaling and antioxidant responses. The reduction in fibrosis-related markers observed in this study is likely secondary to attenuation of oxidative stress and inflammatory signaling, rather than a direct antifibrotic effect of PlGF itself [5]. Consistent with this interpretation, the role of PlGF in fibrosis appears to be context-dependent, and its function in ovarian tissue remains incompletely defined [16].

From a translational perspective, PD-MSC-based approaches may have potential for ovarian conditions associated with metabolic imbalance, oxidative stress, and impaired folliculogenesis, including PCOS and ovarian aging [1,49]. The PlGF/Flt-1 axis may also represent a potential target for antioxidant-oriented interventions. However, several challenges remain, including variability in cell homing efficiency, potential immunogenicity, long-term safety, and scalability of clinical-grade cell production. Addressing these issues will require further studies to evaluate therapeutic mechanisms, establish standardized quality control criteria, and ensure reproducibility and safety in clinical applications [50].

Despite the strengths of the present study, several limitations should be considered. First, PD-MSC transplantation was evaluated at a single dose and time point. These conditions were selected based on previously optimized protocols demonstrating metabolic effects following intravenous administration of 2 × 10^6^ PD-MSCs [25,32]. However, additional dose–response and time-course studies will be required to define the optimal therapeutic window. Second, some experimental endpoints exhibited relatively large variance, reflecting inherent anatomical limitations of the rat ovary. Because each animal provides only two ovaries, tissues were allocated separately for molecular and histological analyses, resulting in differences in effective sample size across assays. Third, this study was conducted using a rat model, and species-specific differences in ovarian physiology, endocrine regulation, and metabolic responses may limit the direct translation of these findings to humans. Furthermore, the in vitro experiments were performed using the KGN cell line, a human granulosa-like tumor-derived cell line, which does not fully recapitulate the characteristics of primary human granulosa cells. These limitations should be considered when interpreting the results.

Future studies incorporating expanded cohorts, primary human cell systems, and clinically relevant models will be necessary to further strengthen statistical power and translational relevance.

**Figure 8:**
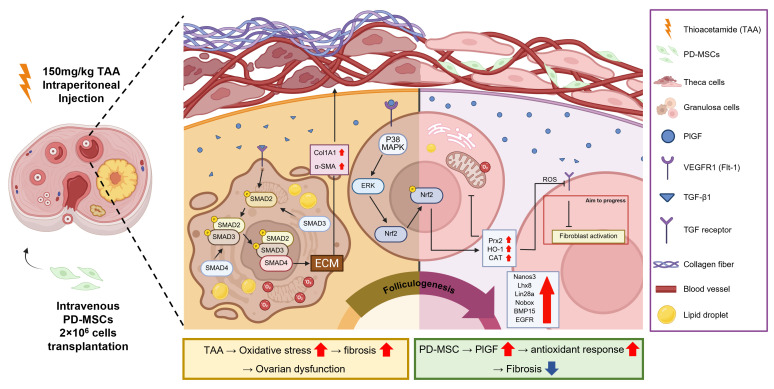
Schematic of the experimental model and proposed mechanism. TAA administration induces ovarian dysfunction characterized by oxidative stress, fibrosis, extracellular matrix (ECM) accumulation, lipid deposition, and impaired follicular development. PD-MSC transplantation is associated with activation of PlGF/Flt-1 signaling and downstream p38 MAPK/ERK/Nrf2 pathways, accompanied by enhanced antioxidant responses and attenuation of fibrosis-related features. A simplified schematic summary is included to facilitate interpretation of the overall mechanism. Created with BioRender. Lee, D. (2026) https://BioRender.com/kvll0fn (accessed on 7 April 2026).

In summary, this study shows that activation of PlGF/Flt-1-related signaling occurs in parallel with antioxidant responses and changes in ovarian structure and redox balance in a TAA-induced ovarian dysfunction model. These findings support a contributory role for PlGF-associated signaling in PD-MSC-mediated redox regulation, rather than a single deterministic mechanism, and provide a framework for future mechanistic and translational studies. A schematic summary of the proposed mechanism is presented in Figure 8.

## 5. Conclusions

In conclusion, this study demonstrates that PD-MSC transplantation is associated with improvement in ovarian structure and redox balance in a TAA-induced ovarian dysfunction model, accompanied by restoration of systemic metabolic balance, partial normalization of endocrine parameters, and enhanced follicular integrity. Our findings indicate that activation of PlGF/Flt-1–related signaling occurs in parallel with antioxidant and cytoprotective responses, including modulation of the p38 MAPK/ERK/Nrf2 axis and increased expression of antioxidant enzymes such as Prx2, HO-1, and Catalase. Our study provides a mechanistic and conceptual framework for future studies exploring stem cell–based or PlGF-oriented strategies to support ovarian structural and redox homeostasis under conditions of metabolic imbalance, oxidative stress, and reproductive aging.

## Figures and Tables

**Table 1:** Follicle counts by developmental stage across experimental groups.

	Primordial	Primary	Secondary	Antral	Atresia
Nor (*n* = 6)	484.72 ± 21.37	78.89 ± 12.40	62.17 ± 12.42	73.13 ± 13.10	57.42 ± 7.46
NTx (*n* = 8)	234.42 ± 19.62 *	46.16 ± 4.64 *	26.75 ± 6.25 *	39.53 ± 3.75 *	142.13 ± 14.22 *
Tx (*n* = 6)	412.21 ± 21.08 **	73.00 ± 10.79 **	48.71 ± 6.32 **	68.96 ± 5.92 **	75.04 ± 8.56 **

*: Nor vs. NTx (*p* < 0.05); **: NTx vs. Tx (*p* < 0.05).

## Data Availability

The original contributions presented in this study are included in the article/Supplementary Material. Further inquiries can be directed to the corresponding authors.

## Supplementary Materials

The following supporting information can be downloaded at: https://www.mdpi.com/article/10.3390/antiox15050603/s1. Supplementary Figure S1. Evaluation of fibrosis markers in ovarian tissue. (a) The mRNA expression of α-smooth muscle actin (α-SMA) was analyzed by qRT-PCR. (b) The mRNA expression of Col1a1 was analyzed by qRT-PCR. The rats were divided into three groups: the Nor (control), NTx (TAAinjured), and Tx (TAA-induced injury and PD-MSC transplantation) groups. Data are presented as the mean ± SE and were analyzed by the Kruskal-Wallis test followed by Conover’s post hoc test with the Benjamini-Hochberg correction. ** *p* < 0.01, *** *p* < 0.001. Supplementary Figure S2. Assessment of mitochondrial membrane potential using TMRE staining. (a) Mitochondrial membrane potential in cells was analyzed by TMRE staining and observed using fluorescence microscopy. Representative images of transmitted light (TRANS), TMRE fluorescence (MMP, red), and merged images (MERGE) are shown. Scale bar = 100 μm. (b) Quantification of TMRE fluorescence intensity was performed using ImageJ software and expressed as relative ΔΨm (fold). Con indicates untreated control cells, TAA indicates TAA-treated cells, Co-cul. indicates TAA-treated and coculture with PD-MSCs, and FCCP indicates FCCP-treated cells as a positive control for mitochondrial depolarization. Data are presented as the mean ± SE and were analyzed by the Kruskal-Wallis test followed by Conover’s post hoc test with the Benjamini-Hochberg correction. *** *p* < 0.001, **** *p* < 0.0001. Supplementary Figure S3. PD-MSC-derived PlGF modulates Flt-1 and downstream signaling pathways in KGN cells. (a) Protein expression of Flt-1 in KGN cells treated with conditioned media (CM) derived from control or siPlGF-transfected PD-MSCs following TAA treatment (b,c) Protein expression of phosphorylated and total p38 MAPK (b) and ERK (c) Relative expression levels are presented as fold changes normalized to GAPDH or total protein levels. KGN cells were treated with conditioned media derived from control PD-MSCs (CM) or siPlGF-transfected PD-MSCs (CM + siRNA) following TAA treatment. Data are presented as the mean ± SEM and were analyzed using the Kruskal–Wallis test followed by Conover’s post hoc test with Benjamini–Hochberg correction. Supplementary Table S1. Primer Sequences for quantitative real-time PCR.
