# Supplementary material for: Placental Growth Factor Secreted from Placenta-Derived Mesenchymal Stem Cells Improves Ovarian Function in TAA-Injured Rats via Antioxidant Effects
Source: Antioxidants (Basel). 2026 May 10;15(5):603. doi: 10.3390/antiox15050603 (PMC13203319; doi:10.3390/antiox15050603)
Supplement: Supplementary file 1 [file antioxidants-15-00603-s001.zip › antioxidants-4266857-supplementary.pdf]

**Antioxidants-4266857**

**Supplementary Table S1. Primer Sequences for quantitative real-time PCR**

| Gene | Primer | Annealing Temperature (℃) |
|---|---|---|
| *Rat TGF-β1* | F: 5'-GAC CTG GGT TGG AAG TGG AT-3' | 58.3 |
| | R: 5'-TCT CCA CAT CAC TAG CTC TCC T-3' | 58.8 |
| *Rat α-SMA* | F: 5'-CGA TAG AAC ACG GCA TCA TCA C-3' | 58.6 |
| | R: 5'-GCA TAG CCC TCA TAG ATA GGC A-3' | 58.2 |
| *Rat Col1a1* | F: 5'-TGG GCA TCT GCT TTA GCC TC-3' | 59.1 |
| | R: 5'-TTC GAT TGT CTT GCC CC-3' | 59.1 |
| *Rat HO-1* | F: 5'-TGC ACA TCC GTG CAG AGA AT-3' | 59.1 |
| | R: 5'-CTG GGT TCT GCT TGT TTC GC-3' | 59.1 |
| *Rat Catalase* | F: 5'-TCA GAG GAA AGC GGT CAA GA-3' | 57.7 |
| | R: 5'-CCC GTG CTT TAC AGG TTA GC-3' | 58 |
| *Rat Nanos3* | F: 5'-CTC TGC ATG AGG AAG AGG AGC C-3' | 61.5 |
| | R: 5'-GGA CTG ATA GAT CGC ACG AGA-3' | 58.2 |
| *Rat Lhx8* | F: 5'-GTA TCA CTT GGC TTG CTT-3' | 51.6 |
| | R: 5'-ATT ACC GTT CTC CAC TTC-3' | 50.2 |
| *Rat Lin28a* | F: 5'-CCC GGT GGA CGT CTT TGT G-3' | 60 |
| | R: 5'-CAC TGC CTC ACC CTC CTT GA-3' | 60.5 |
| *Rat Nobox* | F: 5'-AGC CAG TGC AGA TCT GCA CC-3' | 61.8 |
| | R: 5'-TGT CAC TGC CAG GAA CAT CCC TC-3' | 63.3 |
| *Rat BMP15* | F: 5'-ATC TGA TGT CCC TTG TCC TT-3' | 54.8 |
| | R: 5'-CTC TGT ATG GAT GGC ATG GTT-3' | 54.2 |
| *Rat EGFR* | F: 5'-AGA TTG CAA AGG GCA TGA ACT AC-3' | 58.6 |
| | R: 5'-ACA TTC CTG GCT GCC AAG TC-3' | 59.6 |
| *Rat GAPDH* | F: 5'-TCC CTC AAG ATT GTC AGC AA-3' | 55.8 |
| | R: 5'-AGA TCC ACA ACG GAT ACA TT-3' | 53.2 |

| Gene | Primer | Anneling Teamperature (℃) |
|---|---|---|
| *Human PlGF* | F: 5'-GTT CAG CCC ATC CTG TGT CT-3' | 58.7 |
| | R: 5'-CTT CAT CTT CTC CCG CAG AG-3' | 56.8 |
| *Human Nrf2* | F: 5'-GGT TGC CCA CAT TCC CAA ATC-3' | 59.1 |
| | R: 5'-CAA GTG ACT GAA ACG TAG CCG-3' | 58.6 |
| *Human Catalase* | F: 5'-GTG CGG AGA TTC AAC ACT GCC A-3' | 62.7 |
| | R: 5'-CGG CAA TGT TCT CAC ACA GAC G-3' | 61.3 |
| *Human GAPDH* | F: 5'-GCA CCG TCA AGG CTG AGA AC-3' | 60.6 |
| | R: 5'-GTG GTG AAG ACG CCA GTG GA-3' | 61.4 |

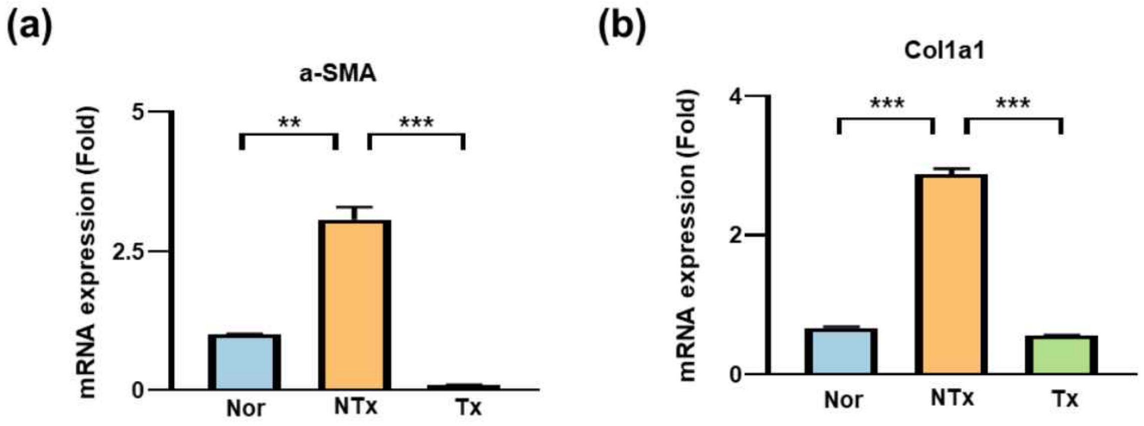


**Supplementary Figure S1.** Evaluation of fibrosis markers in ovarian tissue. **(a)** The mRNA expression of $\alpha$-smooth muscle actin ($\alpha$-SMA) was analyzed by qRT–PCR. **(b)** The mRNA expression of Col1a1 was analyzed by qRT–PCR. The rats were divided into three groups: the Nor (control), NTx (TAA-injured), and Tx (TAA-induced injury and PD-MSC transplantation) groups. Data are presented as the mean ± SE and were analyzed by the Kruskal-Wallis test followed by Conover's post hoc test with the Benjamini-Hochberg correction. ** $p < 0.01$, *** $p < 0.001$.

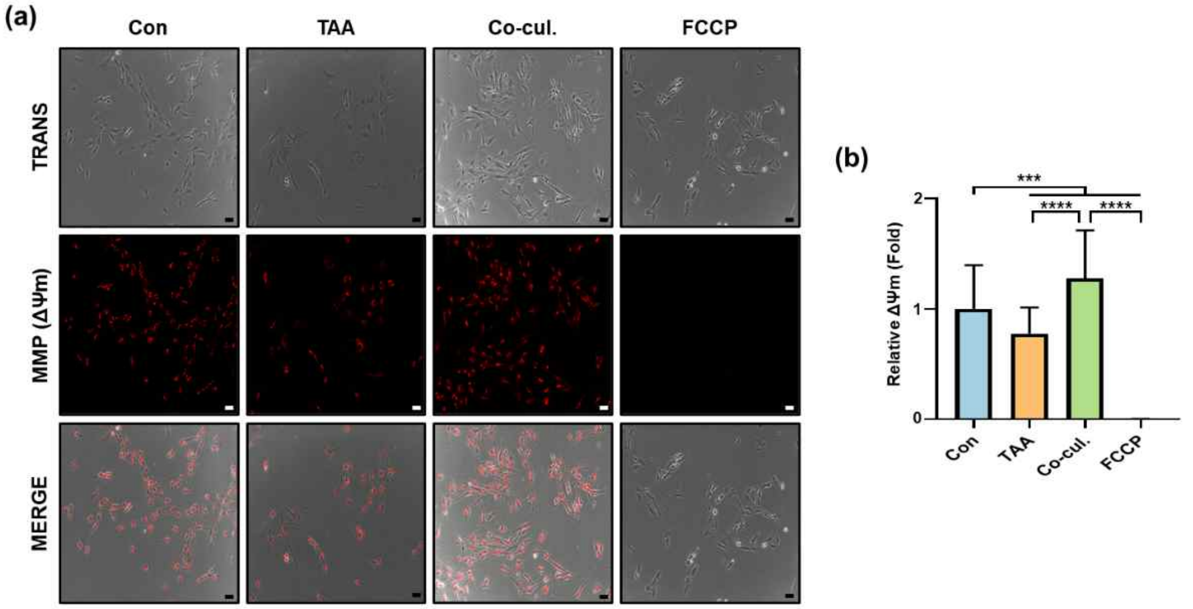


**Supplementary Figure S2.** Assessment of mitochondrial membrane potential using TMRE staining. (a) Mitochondrial membrane potential in cells was analyzed by TMRE staining and observed using fluorescence microscopy. Representative images of transmitted light (TRANS), TMRE fluorescence (MMP, red), and merged images (MERGE) are shown. Scale bar = 100 μm. (b) Quantification of TMRE fluorescence intensity was performed using ImageJ software and expressed as relative ΔΨm (fold). Con indicates untreated control cells, TAA indicates TAA-treated cells, Co-cul. indicates TAA-treated and coculture with PD-MSCs, and FCCP indicates FCCP-treated cells as a positive control for mitochondrial depolarization. Data are presented as the mean ± SE and were analyzed by the Kruskal-Wallis test followed by Conover's post hoc test with the Benjamini-Hochberg correction. *** $p < 0.001$, **** $p < 0.0001$.

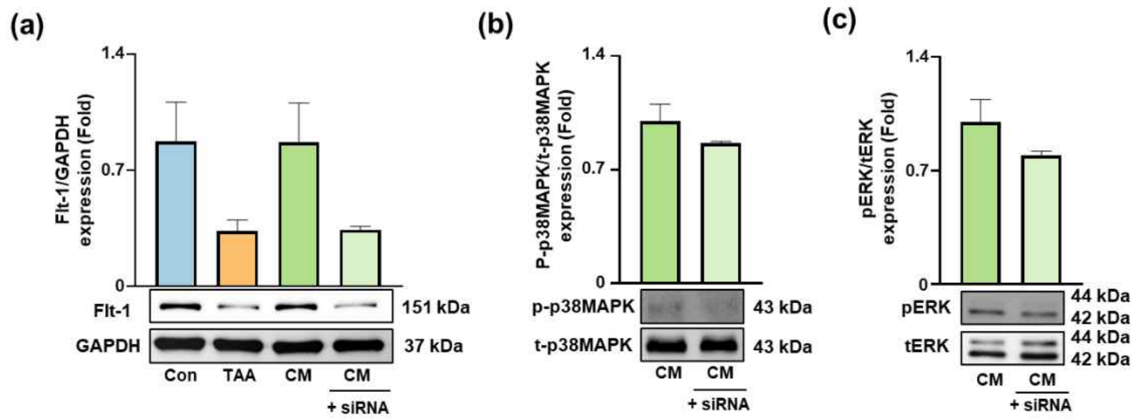


**Supplementary Figure S3.** PD-MSC-derived PlGF modulates Flt-1 and downstream signaling pathways in KGN cells. **(a)** Protein expression of Flt-1 in KGN cells treated with conditioned media (CM) derived from control or siPlGF-transfected PD-MSCs following TAA treatment **(b, c)** Protein expression of phosphorylated and total p38 MAPK **(b)** and ERK **(c)** Relative expression levels are presented as fold changes normalized to GAPDH or total protein levels. KGN cells were treated with conditioned media derived from control PD-MSCs (CM) or siPlGF-transfected PD-MSCs (CM + siRNA) following TAA treatment. Data are presented as the mean ± SEM and were analyzed using the Kruskal–Wallis test followed by Conover's post hoc test with Benjamini–Hochberg correction.
